# *TERT* promoter mutation and chromosome 6 loss define a high-risk subtype of ependymoma evolving from posterior fossa subependymoma

**DOI:** 10.1007/s00401-021-02300-8

**Published:** 2021-03-23

**Authors:** Christian Thomas, Felix Thierfelder, Malte Träger, Patrick Soschinski, Michael Müther, Dominic Edelmann, Alexandra Förster, Carola Geiler, Hee-yeong Kim, Katharina Filipski, Patrick N. Harter, Jens Schittenhelm, Franziska Eckert, Georgios Ntoulias, Sven-Axel May, Walter Stummer, Julia Onken, Peter Vajkoczy, Ulrich Schüller, Frank L. Heppner, David Capper, Arend Koch, David Kaul, Werner Paulus, Martin Hasselblatt, Leonille Schweizer

**Affiliations:** 1 Institute of Neuropathology, University Hospital Münster, Münster, Germany; 2 German Cancer Consortium (DKTK), Partner Site Berlin, German Cancer Research Center (DKFZ), Heidelberg, Germany; 3 Department of Radiation Oncology and Radiotherapy, Charité-Universitätsmedizin Berlin, Corporate Member of Freie Universität Berlin and Humboldt-Universität zu Berlin, Augustenburger Platz 1, 13353 Berlin, Germany; 4 Department of Neurosurgery, University Hospital Münster, Münster, Germany; 5 Division of Biostatistics, German Cancer Research Center, Heidelberg, Germany; 6 Department of Neuropathology, Charité-Universitätsmedizin Berlin, Corporate Member of Freie Universität Berlin and Humboldt-Universität zu Berlin, Charitéplatz 1, 10117 Berlin, Germany; 7 Neurological Institute (Edinger Institute), Goethe University, Frankfurt am Main, Germany; 8 German Cancer Consortium (DKTK), Partner Site Frankfurt/Mainz, German Cancer Research Center (DKFZ), Heidelberg, Germany; 9 Frankfurt Cancer Institute (FCI), Frankfurt am Main, Germany; 10 Department of Neuropathology, Institute of Pathology and Neuropathology, University of Tübingen, Tübingen, Germany; 11 Department of Radiooncology, University Hospital Tübingen, Tübingen, Germany; 12 Department of Neurosurgery, Vivantes Klinikum Neukölln, Berlin, Germany; 13 Department of Neurosurgery, Klinikum Chemnitz, Chemnitz, Germany; 14 Department of Neurosurgery, Charité-Universitätsmedizin Berlin, Corporate Member of Freie Universität Berlin and Humboldt-Universität zu Berlin, Charitéplatz 1, 10117 Berlin, Germany; 15 Department of Neuropathology, University Hospital Hamburg-Eppendorf, Hamburg, Germany; 16 Research Institute Children’s Cancer Center Hamburg, Hamburg, Germany; 17 Department of Pediatric Hematology and Oncology, University Medical Center Hamburg-Eppendorf, Hamburg, Germany; 18 Cluster of Excellence, NeuroCure, Charitéplatz 1, 10117 Berlin, Germany; 19 German Center for Neurodegenerative Diseases (DZNE) Berlin, 10117 Berlin, Germany

**Keywords:** Subependymoma, Mixed ependymoma–subependymoma, Chromosome 6, *TERT*, DNA methylation

## Abstract

Subependymomas are benign tumors characteristically encountered in the posterior fossa of adults that show distinct epigenetic profiles assigned to the molecular group “subependymoma, posterior fossa” (PFSE) of the recently established DNA methylation-based classification of central nervous system tumors. In contrast, most posterior fossa ependymomas exhibit a more aggressive biological behavior and are allocated to the molecular subgroups PFA or PFB. A subset of ependymomas shows epigenetic similarities with subependymomas, but the precise biology of these tumors and their potential relationships remain unknown. We therefore set out to characterize epigenetic traits, mutational profiles, and clinical outcomes of 50 posterior fossa ependymal tumors of the PFSE group. On histo-morphology, these tumors comprised 12 ependymomas, 14 subependymomas and 24 tumors with mixed ependymoma–subependymoma morphology. Mixed ependymoma–subependymoma tumors varied in their extent of ependymoma differentiation (2–95%) but consistently exhibited global epigenetic profiles of the PFSE group. Selective methylome analysis of microdissected tumor components revealed CpG signatures in mixed tumors that coalesce with their pure counterparts. Loss of chr6 (20/50 cases), as well as *TERT* mutations (21/50 cases), were frequent events enriched in tumors with pure ependymoma morphology (*p* < 0.001) and confined to areas with ependymoma differentiation in mixed tumors. Clinically, pure ependymoma phenotype, chr6 loss, and *TERT* mutations were associated with shorter progression-free survival (each *p* < 0.001). In conclusion, our results suggest that subependymomas may acquire genetic and epigenetic changes throughout tumor evolution giving rise to subclones with ependymoma morphology (resulting in mixed tumors) that eventually overpopulate the subependymoma component (pure PFSE ependymomas).

**Supplementary Information:**

The online version contains supplementary material available at 10.1007/s00401-021-02300-8.

## Introduction

Ependymal tumors are central nervous system neoplasms that originate from the wall of the ventricular system along the entire cranio-spinal axis. Among them, subependymomas are slowly growing tumors corresponding to WHO grade I that predominantly arise in the posterior fossa of adults [13]. Long-term outcome is excellent even after subtotal resection [2]. In contrast, ependymomas of the posterior fossa correspond to WHO grades II or III and show a more aggressive clinical course often requiring adjuvant therapy [26]. DNA methylation profiles, gene expression signatures, and cytogenetic characteristics separate posterior fossa ependymal tumors into three distinct molecular subgroups, i.e. “posterior fossa group A” (PFA), “posterior fossa group B” (PFB) and “subependymoma, posterior fossa” (PFSE) [5, 17]. Subependymomas are invariably classified as PFSE [17, 26], whereas the majority of WHO grade II and III ependymomas belong to the molecular subgroups PFA and PFB [17]. PFA ependymomas typically arise in pediatric patients and show few chromosomal alterations, whereas ependymomas of the PFB subgroup occur in older children or adults and harbor extensive chromosomal defects [16, 26]. Taking advantage of large patient cohorts, recent molecular and clinical investigations have demonstrated considerable heterogeneity within PFA [16] and PFB [7] ependymomas, whereas PFSE tumors are generally assumed to represent a rather homogeneous group with favorable outcome [17, 26]. Rare tumors with mixed histological features of ependymoma and subependymoma have repeatedly been recorded, and a small series of mixed tumors has been classified as PFSE [6]. Moreover, a subset of morphologically pure WHO grade II (and some WHO grade III) ependymomas show epigenetic similarities with subependymomas and it has been speculated that these tumors might also be associated with favorable outcomes [17, 26]. Little is known about the biology of these tumors, their potential relationships, as well as clinically useful biomarkers. We, therefore, set out to elucidate epigenetic relationships, mutational profiles, and clinical outcomes of 50 posterior fossa ependymal tumors of the PFSE group.

## Materials and methods

### Histopathology and clinical data acquisition

Formalin-fixed paraffin-embedded samples of 50 ependymal tumors of the methylation class “subependymoma, posterior fossa” (14 subependymomas, 12 ependymomas and 24 cases of mixed ependymoma–subependymoma) were collected from the archives of the Institutes of Neuropathology in Berlin, Münster, Frankfurt, Tübingen, and Hamburg. Follow-up information could be retrieved retrospectively from medical records and treating physicians for 49 cases (98%). Investigations were approved by the Münster ethics committee (2019-638-f-s) and the Charité ethics committee (EA1/077/20).

### DNA methylation profiling

After DNA isolation from formalin-fixed paraffin-embedded tumor samples, purification and bisulfite conversion using standard protocols provided by the manufacturer. Samples were analyzed using the MethylationEPIC BeadChip or HumanMethylation450 array (Illumina Inc., San Diego, CA). Raw IDAT files from both array types (450 k or EPIC) were loaded into the R environment (v3.6.3) using the combineArrays function of the minfi package (v1.32). The getSnpBeta function was used to retrieve beta values of 59 SNP probes located on both arrays. Pairwise sample-to-sample Pearson correlation was plotted with the pheatmap package (v1.0.12) and manual inspection did not indicate evidence for sample mix-up. Methylation-based classification was performed using the Heidelberg Brain Tumor Classifier (version 11b4) [5]. The following filtering criteria were applied: removal of probes targeting the X and Y chromosomes, removal of probes containing a single nucleotide polymorphism (dbSNP132 Common) within five base pairs of and including the targeted CpG-site, and probes not mapping uniquely to the human reference genome (hg19) allowing for one mismatch. For comparison, previously published DNA methylation profiles of the Heidelberg Brain Tumor Classifier cohort (GEO accession number GSE90496) [5] were evaluated. Unsupervised t-Distributed Stochastic Neighbor Embedding (t-SNE) analysis across the whole dataset was performed as previously described [22] using the Rtsne package (version 0.15) with the following parameter adjustments: pca = F, theta = 0, max_iter = 2500. Pairwise Pearson correlation was calculated for the 9002 most variable methylation probes (standard deviation > 0.2) across the whole dataset using the wtd.cors function of the weights package (version 1.0). Pairwise sample distances were calculated using 1 minus the weighted Pearson correlation coefficient as the distance measure. The resulting distance matrix was used to perform the clustering analysis. Samples were clustered using the Euclidean distance as the distance measure and Ward’s linkage method. Copy-number variation analysis was performed using the conumee package (http://www.bioconductor.org/packages/release/bioc/html/conumee.html). Chromosomal gains and losses were examined by manual inspection of each profile. Methylation profiles of normal brainstem tissue (*n* = 12 samples) were obtained from GEO (accession number GSE90496) and differential methylation analyses were performed using the limma package (version 3.46). Methylation data were deposited at the public repository Gene Expression Omnibus under the accession number GSE169265.

### Next-generation sequencing

Targeted next-generation sequencing was performed on 12 ependymal tumors (1 subependymoma, 8 mixed subependymomas/ependymomas and 3 ependymomas) using the INVIEW Oncopanel All-in-one, a hybridization-based target capture panel based on Agilent SureSelect technology covering 591 cancer-specific genes. The libraries were sequenced on an Illumina (San Diego, CA) platform at Eurofins Genomics, Ebersberg, Germany.

### Variant calling

Adapter trimming of raw fastq files from targeted panel sequencing was performed using trimmomatic v0.39. Sequences were aligned to the hg38 human reference genome (GRCh38_full_analysis_set_plus_decoy_hla) with the Burrows-Wheeler Aligner algorithm (v0.7.17). Base quality score recalibration was performed using the GATK v4.1.4 suite. Duplicate reads were removed using sambamba v0.7.0. Samtools v1.1.0 was used for BAM file handling. Variant calling for SNVs and indels was performed using Platypus v0.8.1.2 [18]. In addition to hard filtering, only variants at positions with a minimum coverage of 30 reads and > 15% SNV or indel variant reads, respectively, were considered. Variants were annotated using the variant effect predictor (v98.3) for functional annotations, pathogenicity scores, population allele frequencies (1000 Genomes, gnomeAD and ESP6500) and to determine the effect of called variants on genes, transcripts, and protein sequence. Variants were further annotated using the CancerVar script (https://www.github.com/WGLab/CancerVar) to classify each variant into the three categories “likely benign/benign”, “uncertain significance” and “likely pathogenic/pathogenic” according to the joint AMP-ASCO-CAP 2017 guidelines for cancer variant interpretation (22). Custom in-house R and Python scripts were used for a filtering strategy to select for non-synonymous variants in coding regions with a maximum population allele frequency of less than 1%. Variants occurring in ≥ 5 samples were visually inspected for their validity and discarded if likely artifacts. Variants with the ClinVar annotation “benign” or “likely benign” as well as variants with the CancerVar verdict “likely benign/benign” were discarded. The *TERT* promoter region was manually inspected using the integrative genomics viewer (IGV).

### Sanger sequencing

The *TERT* promoter mutations (hg19 genomic position chr5:1295228 and chr5:1295250) were evaluated using Sanger Sequencing (forward primer: GGATTCGCGGGCACAGAC; reverse primer: CAGCGCTGCCTGAAACTC; details on PCR conditions is available upon request). Sequencing was performed at Eurofins Genomics, Ebersberg, Germany.

### Statistical analysis

Patient characteristics were summarized using median and interquartile range (IQR) for continuous variables and percentage for categorical variables. Progression-free survival (PFS) was defined as the time interval between initial surgery and evidence of tumor progression on follow-up magnetic resonance imaging (MRI). Overall survival (OS) was defined as the time from the date of diagnosis to the date of death. Survival analysis was performed using Kaplan–Meier estimation for survival curves and the log-rank test using the survminer R package (version 0.4.8). *p* < 0.05 was considered statistically significant.

## Results

### DNA methylation profiles classify mixed ependymoma–subependymomas as PFSE

**Fig. 1:**
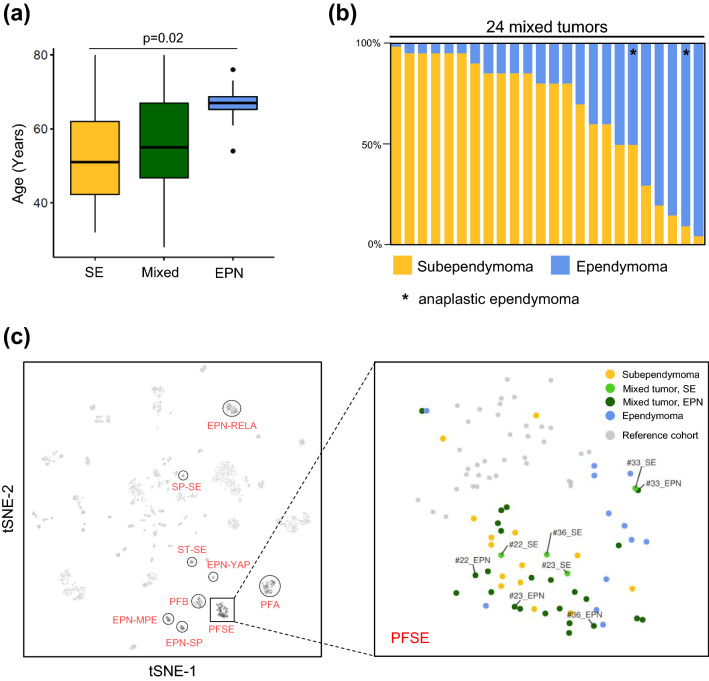
Clinical data, histology and DNA methylation profiles. **a** Age distribution across histological subgroups. **b** Percentage of areas with subependymoma and ependymoma differentiation in mixed tumors. **c** Unsupervised t-SNE analysis of all 50 PFSE plus 4 spatially sampled mixed tumors together with 2801 samples comprising 82 distinct molecular CNS tumor entities. Established ependymal tumor subgroups are indicated in red

We profiled the DNA methylomes of 50 posterior fossa ependymal tumors comprising 14 pure subependymomas, 12 pure ependymomas and 24 mixed tumors with features of both subependymoma and ependymoma (Table S1). Patients with pure ependymomas were older (mean age 67 years) compared to mixed ependymoma–subependymomas (mean age 55 years) and subependymomas (mean age 51 years) (ANOVA, *p* = 0.02; Fig. 1a; Table S1). The histological composition of mixed ependymoma–subependymomas was highly variable, ranging from 2% ependymoma area to 95% ependymoma area (Fig. 1b). In 2/24 mixed tumors, the ependymoma component was characterized by high cellular density, brisk mitotic activity and/or microvascular proliferation corresponding to anaplastic ependymoma (WHO grade III). DNA methylation profiles were obtained from the ependymoma component in all 24 mixed tumors and in a subset of four tumors, both spatially separated components were analyzed. Using DNA methylation-based classification (Heidelberg Brain Tumor Classifier version v11b4) [5] followed by t-distributed stochastic neighbor embedding (t-SNE) analysis together with a reference cohort of 2801 methylation profiles (comprising 2682 CNS tumors and 119 non-neoplastic samples) [5], all samples could be assigned to the molecular PFSE group (Fig. 1c, Table S1), suggesting similar global DNA methylation signatures. We next sought to perform a more focused analysis of microdissected tumor components of mixed ependymoma–subependymomas.

### Methylomes of ependymoma and subependymoma components show distinct signatures

**Fig. 2:**
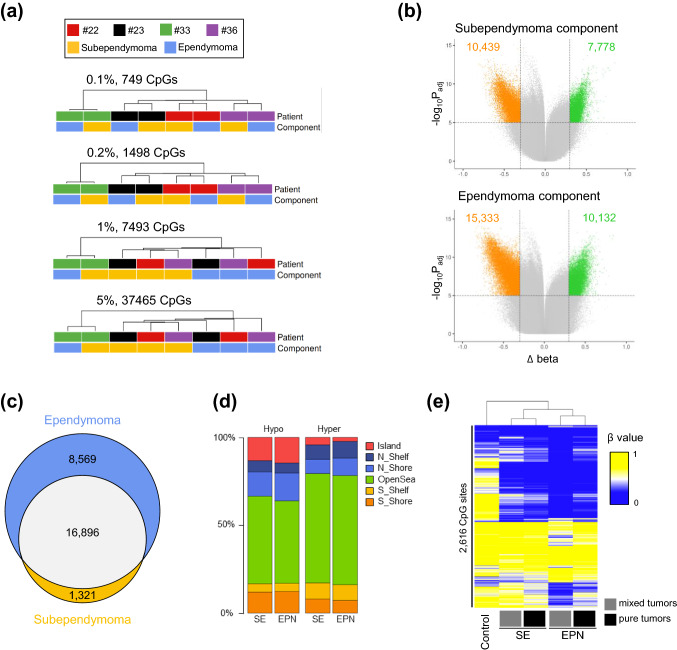
Characteristics of the methylome of ependymoma and subependymoma components. **a** Unsupervised hierarchical clustering of the top 0.1%, 0.2%, 1% and 5% most variable CpG sites. Annotations of sample type and patient identification numbers are provided. **b** The average methylation change from subependymoma components to normal brain controls (top) and ependymoma to normal brain controls (bottom). Colored dots represent CpG sites that show significant hypomethylation (orange dots, total count provided) or hypermethylation (green dots, total numbers provided) at each tumor component (*p* value_adjusted_ < 0.05 and |∆*β*|> 0.3). **c** Venn diagram showing overlap and unique CpG sites for each component. **d** Fractions of hypo- and hypermethylated unique CpG sites of subependymoma (SE) and ependymoma (EPN) components compared to normal brain stem tissue within different epigenomic substructures. **e** Unsupervised hierarchical clustering of 2616 differentially methylated CpG sites between the ependymoma and subependymoma component (*p* value_adjusted_ < 0.05). Heatmap shows average *β* values of brain stem control samples (*n* = 12), subependymoma and ependymoma component of mixed tumors (each *n* = 4), and pure subependymomas (*n* = 14) and ependymomas (*n* = 12)

To determine the extent to which mixed tumor components have altered methylomes beyond the ubiquitous PFSE methylation patterns, we identified the most variable CpG sites across four separately profiled mixed tumors and performed unsupervised hierarchical clustering (probes targeting sex chromosomes and single nucleotide polymorphisms were excluded). When using a strict cut-off value (0.1% most variable CpGs), subependymoma and ependymoma components from the same individual clustered together (Fig. 2a). This result potentially reflects patient-specific methylation patterns, consistent with previous reports on gliomas [12, 14], and maybe indicative of normal inter-individual epigenetic variation, patient-specific methylation characteristics from different disease stages, or both. Progressively applying a higher tolerance in the selection of variable CpG sites (0.2%, 1% and 5%), a gradual switch in clustering patterns was evident with ependymoma and subependymoma components of #22, #23, and #36 clustering together (Fig. 2a). In line with t-SNE analysis (Fig. 1c), both components of #33 remained separated from the other samples, suggesting a strong patient-specific methylome signature.

DNA methylation differences between a normal brain and primary central nervous system tumors may reflect a combination of somatically acquired epigenetic changes and differences between the normal brain tissue and the tumor’s cell of origin [10, 23, 27]. Therefore, we separately compared differentially methylated CpG sites of the subependymoma and ependymoma components, respectively, with non-neoplastic tissue of the brain stem (pons). Using this approach, we were able to identify 18,217 differentially methylated CpG sites in the subependymoma component and 25,465 differentially methylated CpG sites in the ependymoma component (Fig. 2b). There was a striking overlap of differentially methylated CpG sites in both tumor components (Fig. 2c). We, therefore, hypothesized that these CpGs may reflect differences between the normal brain and the tumor’s cell of origin. The subependymoma component showed a higher proportion of overlapping CpGs (16,896/18,217 CpG sites, 93%) compared to the ependymoma component that showed a considerable fraction of private CpGs (8569/25,465 CpG sites, 34%), presumably reflecting the continuous accumulation of methylation alterations throughout tumor progression associated with a histological ependymoma phenotype. The fraction of probes changing their methylation in the different epigenomic substructures was very similar between private CpGs of each component (Fig. 2d).

### Distinct methylation signatures of mixed PFSE tumors components associate with their pure counterparts

Next, we calculated differentially methylated CpG sites (adj. *p* value < 0.05) when directly comparing the ependymoma and subependymoma components of mixed tumors. Hierarchical clustering of the resulting 2616 CpG sites demonstrated a methylation signature where the subependymoma component of mixed tumors clustered with pure subependymomas and the ependymoma component of mixed tumors clustered with pure ependymomas of our cohort, respectively (Fig. 2e).

Taken together, these analyses suggest that the ependymoma and subependymoma components of mixed PFSE tumors contain a methylation signature that coalesces with their pure counterparts. We, therefore, speculated that the ependymoma component arises throughout tumor evolution and sought to dissect genetic changes in PFSE tumors that are involved in tumor progression.

### Copy-number variation and DNA sequencing analysis reveal recurrent loss of chromosome 6 and *TERT* promoter mutations, both associated with global DNA methylation patterns

**Fig. 3:**
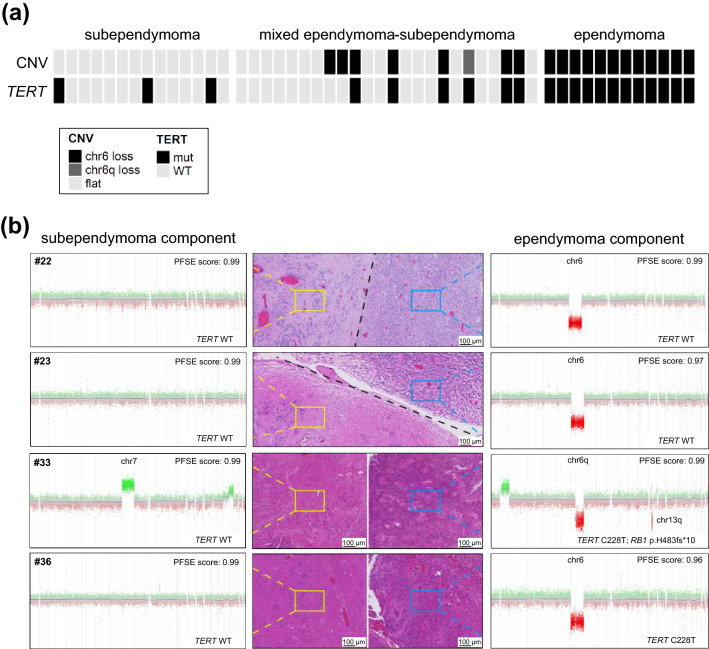
*TERT* promoter mutations and copy-number alterations in 50 PFSE tumors. **a** *TERT* promoter mutation status and CNV status in all 50 PFSE tumors. **b** CNV profiles derived from microdissected tumor components in four mixed ependymoma–subependymomas (#22, #23, #33 and #36). PFSE scores indicate the methylation-based classification score of the DKFZ brain tumor classifier (v11b4)

Copy-number analysis was performed using signal intensity values of DNA methylation profiles [5]. The majority of tumors (26/50, 52%) showed a balanced copy-number variation (CNV) profile. Copy-number alterations mainly comprised whole-chromosomal or chromosomal-arm alterations (Table S1). The most frequently observed copy-number alteration was a loss of whole chromosome 6 (19/50 cases, 38%; Fig. 3a). In addition, loss of chromosome 6q was encountered in one mixed ependymoma–subependymoma. Alterations of chromosome 6 were significantly associated with histological subgroups: 0/14 subependymomas, 8/24 (33%) mixed ependymoma/subependymomas, 12/12 (100%) ependymomas (Chi-square, *p* = 9 × 10^–7^). Loss of whole chromosome 8 was observed in three tumors (6%). Gain of chromosome 1q, for which a prognostic role has been demonstrated in ependymomas [17], was present in only 1/50 cases (2%). In four mixed ependymoma–subependymoma tumors, separate copy-number profiles of both histological components revealed that loss of chromosome 6 was confined to the ependymoma component (Fig. 3b).

Targeted next-generation sequencing was performed in one subependymoma, eight mixed ependymoma–subependymomas and three ependymomas (Table S1). According to our filter criteria (see methods), a total of 37 non-synonymous single nucleotide variants (SNVs) and small-scale somatic insertion/deletions (indels) in coding regions were identified (Table S2). All variants were manually curated and further classified according to the five-tier system of the American College of Medical Genetics and Genomics (ACMG) resulting in 17/37 (46%) likely benign/benign variants, 15/37 (41%) variants of uncertain significance, 4/37 (11%) likely pathogenic variants and only one pathogenic variant occurring in case #33, a mixed tumor composed of anaplastic ependymoma and subependymoma (Fig. 3b, Table S2). This pathogenic *RB1* missense variant affects exon 16 (NM_000321, c.1442dupT) resulting in a frameshift mutation (p.H483Sfs*10). Of note, this tumor also harbored a focal loss affecting chr13q14.2 (including *RB1*; Fig. 3b), that was only present in the ependymoma component, suggesting biallelic inactivation of *RB1*. The four likely pathogenic variants comprise missense variants in the *PTPRD* and *TSC2* tumor suppressor genes [#34 and #42: *PTPRD* exon14:c.T2243A:p.L748Q (NM_001171025) and #38 and #40 *TSC2* exon2:c.G76A:p.E26K (NM_001318829)], but variant allele frequencies around 50% suggest heterozygous germline mutations without evidence of copy-number alteration affecting the other allele in those cases.

Inspection of the *TERT* promoter revealed C228T mutations in four samples with sufficient coverage (#31, #33, #40, and #42). This prompted us to perform Sanger sequencing of the *TERT* promoter in the remaining cases confirming hotspot mutations in 21/50 (42%) tumors (two samples with C250T and 19 samples with C228T mutations). *TERT* promoter mutations were enriched in tumors with ependymoma phenotype: all 12/12 (100%) ependymomas showed a mutation as compared to 6/23 (26%) mixed ependymomas-subependymomas and 3/13 (23%) subependymomas (Chi-square, *p* = 3.4 × 10^–5^). Patients with *TERT* promoter mutations were significantly older (mean age 64 years) as compared to *TERT* wild type (mean age 52 years) (*t* test, *p* = 0.0012). In two mixed ependymoma–subependymoma samples (cases #33 and #36), spatial *TERT* sequencing revealed a C228T mutation in the ependymoma component and wild type sequences in the subependymoma component (Fig. 3b).

### Loss of chromosome 6 and *TERT* promoter mutations are associated with global DNA methylation patterns

We next sought to analyze if genetic alterations are associated with global DNA methylation signatures in PFSE tumors. Unsupervised hierarchical clustering of all samples revealed two major methylation clusters (Figure S1). Cluster 1 is comprised of 13 subependymomas and 16 mixed tumors, whereas cluster 2 contains all 12 pure ependymomas, 8 mixed tumors and 1 subependymoma. Of note, tumors with chromosome 6 loss (*n* = 20) were exclusively encountered in methylation cluster 2 (Chi-square, *p* < 0.001). Moreover, methylation cluster 2 contained the vast majority of *TERT* mutated PFSE tumors (19/21, 90%, Chi-square *p* < 0.001).

### Pure ependymoma phenotype, loss of chromosome 6, and *TERT* promoter mutations are associated with tumor progression

**Fig. 4:**
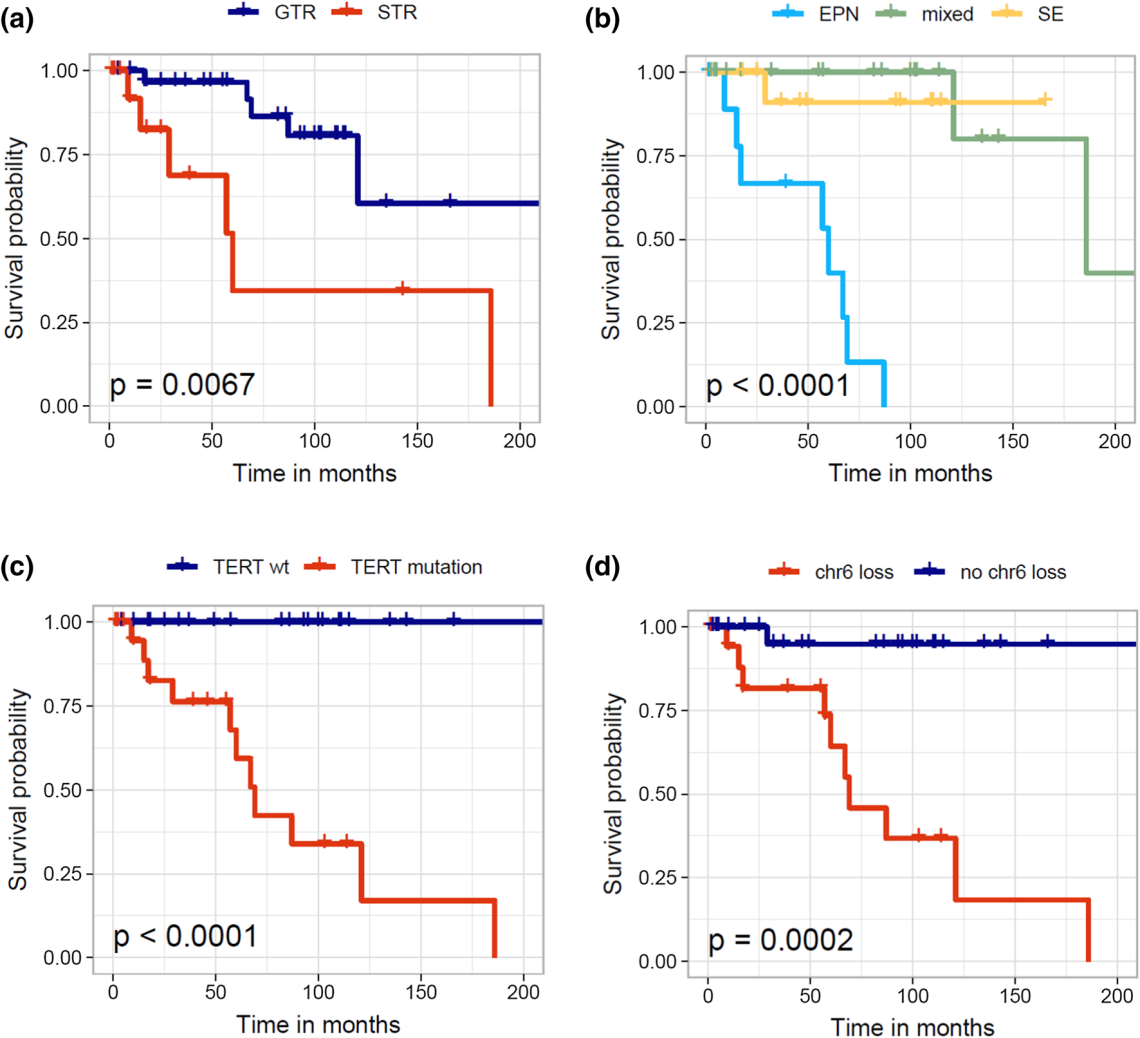
Progression-free survival in 49 PFSE tumors. Subtotal resection (**a**), pure ependymoma morphology (**b**), *TERT* promoter mutation (**c**) and chromosome 6 loss (**d**) are significantly associated with reduced progression-free survival in PFSE tumors

After a median observation period of 55 months, 35 patients were alive without evidence of tumor residuals or stable disease, whereas 11 patients experienced tumor progression and one patient had succumbed to the disease (Table S1, Figure S2). Four patients died for unknown or non-cancer-related reasons and one patient died from postoperative complications and was excluded from survival analysis. Gross total resection (GTR) was achieved in 33/49 patients (67%) (Table S1). Subtotal resection (STR) was significantly more frequent in pure ependymomas (8/12, 67%) as compared to mixed ependymoma–subependymoma tumors (6/23, 26%) and pure subependymomas (2/12, 17%) (Chi-square test, *p* = 0.01). Progression-free survival was significantly shorter in incompletely resected tumors (Log-rank test, *p* = 0.0067; Fig. 4a) and in ependymomas compared to mixed ependymomas-subependymoma tumors and subependymomas (Log-rank test, *p* < 0.0001; Fig. 4b). Of note, *TERT* promoter mutation status (Log-rank test, *p* < 0.0001; Fig. 4c) and chromosome 6 loss (Log-rank test, *p* = 0.0002; Fig. 4) were significantly associated with shorter progression-free survival (Fig. 4d). *TERT* promoter mutation status most clearly segregated progressing and stable tumors and these high-risk PFSE tumors showed significantly worse outcome compared to a previously published series of 137 PFB ependymomas [7] (Figure S3).

## Discussion

We analyzed a series of 50 posterior fossa ependymal tumors of the PFSE group and evaluated histological as well as molecular features and their association with patient outcome. Our study comprised 14 subependymomas, 24 cases of mixed ependymoma–subependymomas with varying proportions of ependymoma differentiation, and 12 pure ependymomas without apparent subependymoma component. Tumors showing features of both ependymoma and subependymoma differentiation have long been on record [2, 20, 21] and raise the question regarding their histogenesis. Some authors have speculated that these two components might reflect a “collision” phenomenon of two separate neoplastic clones [9], whereas alternative hypotheses assume a shared precursor cell with early divergence into distinct subclones (combination phenomenon) or one component reflecting metaplasia or dedifferentiation from another (conversion phenomenon) [1, 24]. Given that cancer DNA methylation profiles represent a combination of somatically acquired DNA methylation changes and a strong signature reflecting the cell of origin [10], it is reasonable to assume that PFSE ependymal tumors share a common precursor cell that acquires additional (subclonal) genetic and epigenetic alterations throughout tumor evolution shaping the histological phenotype. Corroborating this hypothesis, separately dissected methylome signatures of ependymoma and subependymoma components were both clearly classified as PFSE and showed large overlaps of epigenetic alterations when compared to normal brain methylomes. Based on a small subset of CpG sites, we dissected a signature that is specific to each subtype and presumably reflects a subset of acquired somatic methylation changes. Of note, when comparing methylation values based on this signature, supendymoma and ependymoma components of mixed tumors cluster with the signatures of pure subependymomas and ependymomas, respectively. Taken together, notwithstanding different histological phenotypes, global methylome profiles of PFSE tumors strongly suggest a common histogenesis from a shared precursor cell.

Despite epigenetic characteristics, we also analyzed genetic events being associated with specific histo-phenotypes. In line with previous observations [6], loss of chromosome 6 was confined to regions with ependymoma morphology in a set of mixed tumors. Furthermore, we were able to demonstrate that *TERT* promoter mutations exclusively occurred in the ependymoma component in two mixed tumors (#33 and #36). Next-generation sequencing revealed a pathogenic *RB1* mutation within the ependymoma component of case #33 with the other allele being affected by a deletion on chr13, whereas the subependymoma component of the same tumor did not show any chromosomal alterations on chr13 (Fig. 3b). The phenomenon of specific mutations shaping the histological appearance is well known for other brain tumors with mixed morphological phenotypes, such as atypical teratoid/rhabdoid tumors (AT/RT) arising in pleomorphic xanthoastrocytoma or ependymoma with loss of SMARCB1/INI1 expression being confined to the AT/RT component [8, 15]. Further supported by the observation that all 12 ependymomas of our study harbored both a *TERT* promoter mutation and loss of chromosome 6, it is tempting to speculate that these alterations are involved in tumor evolution of subclones with ependymoma morphology, given that we also observed mixed tumors without these alterations in regions with ependymoma differentiation.

**Fig. 5:**
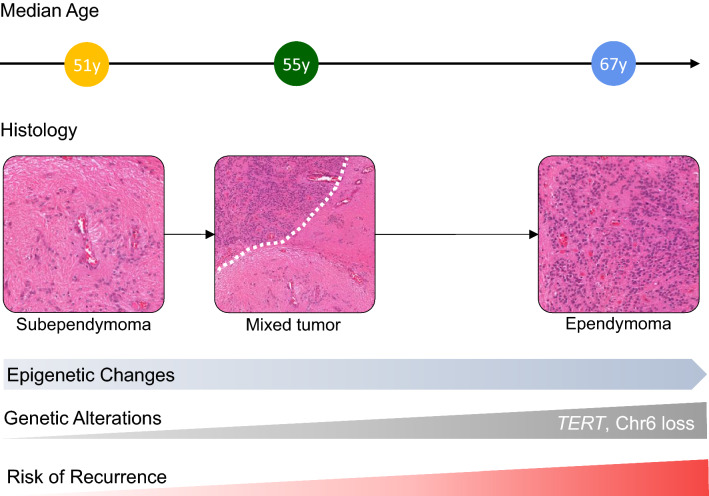
Synopsis of epidemiological, histological, epigenetic, genetic and clinical findings

*TERT* promoter mutations have emerged as denominators of specific subgroups among diffuse astrocytomas with a higher propensity to recur as high-grade tumors and allow for the classification of otherwise WHO grade II diffuse astrocytoma as WHO grade IV [3]. In other cancer types, *TERT* promoter mutations are associated with older age [11, 25]. A recent study evaluating the presence of *TERT* promoter mutations in ependymal tumors across different age groups demonstrated mutations in 9/120 samples (7%) occurring in conventional ependymomas diagnosed in adults [4]. Of note, two of the nine mutant cases recurred with a sarcomatous component being diagnosed as ependymosarcoma and in one patient, microdissection revealed the presence of the *TERT* mutation being confined to the sarcomatous component. Moreover, cases initially diagnosed as subependymoma recurring with atypical features [24] or as ependymoma (case #36 in [2]) are on record. Taken together, the notion that patients with *TERT*-mut/Chr6-loss pure ependymomas in our cohort were significantly older compared to mixed ependymoma–subependymomas and pure subependymomas further corroborates our model where the ependymoma component arises secondarily due to acquired molecular changes over time (Fig. 5).

Historical series suggested that mixed ependymoma–subependymomas behave more aggressively than pure subependymomas [21], although more recent analyses remain controversial about this observation [2, 9, 20]. The current 2016 World Health Organization (WHO) classification [13] recommends to grade mixed ependymoma–subependymoma tumors on the basis of the ependymoma component, although no explicit percentage of ependymoma component is indicated. Our outcome analyses suggest that mixed tumors generally show a good prognosis following tumor resection with only two recurrences. Overall, *TERT*/Chr6-wildtype tumors show a lower propensity of progression compared to *TERT*-mut/Chr6-loss PFSE tumors. Of note, loss of chromosome 6 is not associated with an increased risk of recurrence in PFB tumors [7] and might thus represent a methylation subgroup-specific effect, similar to gain of chromosome 1q which is only prognostically relevant in PFA tumors [7, 16].

Given that pure ependymomas arising in the posterior fossa fall into three clinically relevant molecular groups (PFB, PFSE or, rarely, PFA) that cannot be distinguished by histological features [17], molecular profiling is warranted in these tumors. Whereas PFA ependymomas primarily arise in children and display poor outcome, PFB ependymomas occur in older children and adults and generally show a more favorable outcome [17]. PFSE ependymomas almost exclusively occur in adults and were previously thought to be associated with good outcomes [17]. In contrast, our data suggest that ependymomas of the PFSE subgroup frequently recur and display even worse progression-free survival compared to previously reported PFB ependymomas in adults [26], although this finding might be biased by higher age at diagnosis in our series and needs to be validated in larger cohorts. Currently, the standard of care for all posterior fossa ependymomas in pediatric patients is maximal safe surgical resection followed by radiation therapy, but in adults, the role of adjuvant postoperative radiation is unclear resulting in a subset of patients who are treated with surgery only [19]. Our results suggest that a more aggressive therapy regime might be considered especially in incompletely resected *TERT*-mut/Chr6-loss PFSE tumors. In routine clinical practice, posterior fossa ependymoma patients can be stratified into the established molecular groups PFSE, PFB and PFA using DNA methylation profiling. In addition, DNA copy-number profiles derived from methylation intensity values readily identify PFSE patients with loss of chromosome 6. Since *TERT* mutations only occur in two proximal promoter hotspot regions, targeted testing might serve as an amenable approach to detect high-risk *TERT*-mut/Chr6-loss PFSE tumors.

Taken together, our results indicate that subependymomas, mixed ependymoma–subependymoma tumors, and pure ependymomas of the PFSE group share close epigenetic relationships suggesting a common cellular origin. Our findings suggest that subependymomas represent precursor lesions with the propensity to progress to mixed tumors with an ependymoma phenotype and eventually to pure ependymomas due to acquired genetic and epigenetic changes over time. In PFSE tumors with pure ependymoma phenotype, *TERT*-mutation/Chr6-loss is associated with increased risk of recurrence and these alterations might therefore represent useful markers for more aggressive therapy regimes.

## Supplementary Information

Below is the link to the electronic supplementary material.

Supplementary file1 (PPTX 213 KB)

Supplementary file2 (XLSX 20 KB)

Supplementary file3 (XLSX 13 KB)

## References

[1] Arvanitis LD, Gattuso P, Nag S (2013). A 40-year-old male with an intraventricular tumor. Brain Pathol.

[2] Bi Z, Ren X, Zhang J, Jia W (2015). Clinical, radiological, and pathological features in 43 cases of intracranial subependymoma. J Neurosurg.

[3] Brat DJ, Aldape K, Colman H, Holland EC, Louis DN, Jenkins RB (2018). cIMPACT-NOW update 3: recommended diagnostic criteria for “Diffuse astrocytic glioma, IDH-wildtype, with molecular features of glioblastoma, WHO grade IV”. Acta Neuropathol.

[4] Brügger F, Dettmer MS, Neuenschwander M, Perren A, Marinoni I, Hewer E (2016). TERT promoter mutations but not the alternative lengthening of telomeres phenotype are present in a subset of ependymomas and are associated with adult onset and progression to ependymosarcoma. J Neuropathol Exp Neurol.

[5] Capper D, Jones DTW, Sill M, Hovestadt V, Schrimpf D, Sturm D (2018). DNA methylation-based classification of central nervous system tumours. Nature.

[6] Capper D, Stichel D, Sahm F, Jones DTW, Schrimpf D, Sill M (2018). Practical implementation of DNA methylation and copy-number-based CNS tumor diagnostics: the Heidelberg experience. Acta Neuropathol.

[7] Cavalli FMG, Hübner J-M, Sharma T, Luu B, Sill M, Zapotocky M (2018). Heterogeneity within the PF-EPN-B ependymoma subgroup. Acta Neuropathol.

[8] Chacko G, Chacko AG, Dunham CP, Judkins AR, Biegel JA, Perry A (2007). Atypical teratoid/rhabdoid tumor arising in the setting of a pleomorphic xanthoastrocytoma. J Neurooncol.

[9] Gavankar C, Grant RA, Fulbright R (2015). Mixed tumor with subependymoma and ependymoma features: a case report and review of the literature. J Neurol Neurosci.

[10] Hovestadt V, Jones DTW, Picelli S, Wang W, Kool M, Northcott PA (2014). Decoding the regulatory landscape of medulloblastoma using DNA methylation sequencing. Nature.

[11] Koelsche C, Sahm F, Capper D, Reuss D, Sturm D, Jones DTW (2013). Distribution of TERT promoter mutations in pediatric and adult tumors of the nervous system. Acta Neuropathol.

[12] Laffaire J, Everhard S, Idbaih A, Criniere E, Marie Y, de Reynies A (2011). Methylation profiling identifies 2 groups of gliomas according to their tumorigenesis. Neurooncology.

[13] Louis DN, Ohgaki H, Wiestler ODCW (2016). World Health Organization histological classification of tumours of the central nervous system.

[14] Mazor T, Pankov A, Johnson BE, Hong C, Hamilton EG, Bell RJA (2015). DNA methylation and somatic mutations converge on the cell cycle and define similar evolutionary histories in brain tumors. Cancer Cell.

[15] Nobusawa S, Hirato J, Sugai T, Okura N, Yamazaki T, Yamada S (2016). Atypical teratoid/rhabdoid tumor (AT/RT) arising from ependymoma: a type of AT/RT secondarily developing from other primary central nervous system tumors. J Neuropathol Exp Neurol.

[16] Pajtler KW, Wen J, Sill M, Lin T, Orisme W, Tang B (2018). Molecular heterogeneity and CXorf67 alterations in posterior fossa group A (PFA) ependymomas. Acta Neuropathol.

[17] Pajtler KW, Witt H, Sill M, Jones DTW, Hovestadt V, Kratochwil F (2015). Molecular classification of ependymal tumors across all CNS compartments, histopathological grades, and age groups. Cancer Cell.

[18] Rimmer A, Phan H, Mathieson I, Iqbal Z, Twigg SRF, Wilkie AOM (2014). Integrating mapping-, assembly- and haplotype-based approaches for calling variants in clinical sequencing applications. Nat Genet.

[19] Rudà R, Reifenberger G, Frappaz D, Pfister SM, Laprie A, Santarius T (2018). EANO guidelines for the diagnosis and treatment of ependymal tumors. Neurooncology.

[20] Rushing EJ, Cooper PB, Quezado M, Begnami M, Crespo A, Smirniotopoulos JG (2007). Subependymoma revisited: clinicopathological evaluation of 83 cases. J Neurooncol.

[21] Scheithauer BW (1978). Symptomatic subependymoma. Report of 21 cases with review of the literature. J Neurosurg.

[22] Schweizer L, Thierfelder F, Thomas C, Soschinski P, Suwala A, Stichel D (2020). Molecular characterization of CNS paragangliomas identifies cauda equina paragangliomas as a distinct tumor entity. Acta Neuropathol.

[23] Sproul D, Kitchen RR, Nestor CE, Dixon JM, Sims AH, Harrison DJ (2012). Tissue of origin determines cancer-associated CpG island promoter hypermethylation patterns. Genome Biol.

[24] Tiwari N, Powell SZ, Takei H (2015). Recurrent subependymoma of fourth ventricle with unusual atypical histological features: a case report. Pathol Int.

[25] Vinagre J, Almeida A, Pópulo H, Batista R, Lyra J, Pinto V (2013). Frequency of TERT promoter mutations in human cancers. Nat Commun.

[26] Witt H, Gramatzki D, Hentschel B, Pajtler KW, Felsberg J, Schackert G (2018). DNA methylation-based classification of ependymomas in adulthood: implications for diagnosis and treatment. Neuro Oncol.

[27] Witte T, Plass C, Gerhauser C (2014). Pan-cancer patterns of DNA methylation. Genome Med.

