# Supplementary material for: TERT promoter mutation and chromosome 6 loss define a high-risk subtype of ependymoma evolving from posterior fossa subependymoma
Source: Acta Neuropathol. 2021 Mar 23;141(6):959–70. doi: 10.1007/s00401-021-02300-8 (PMC8113189; doi:10.1007/s00401-021-02300-8)
Supplement: Supplementary file 1 — Supplementary file1 (PPTX 213 KB) [file 401_2021_2300_MOESM1_ESM.pptx]

## Slide 1
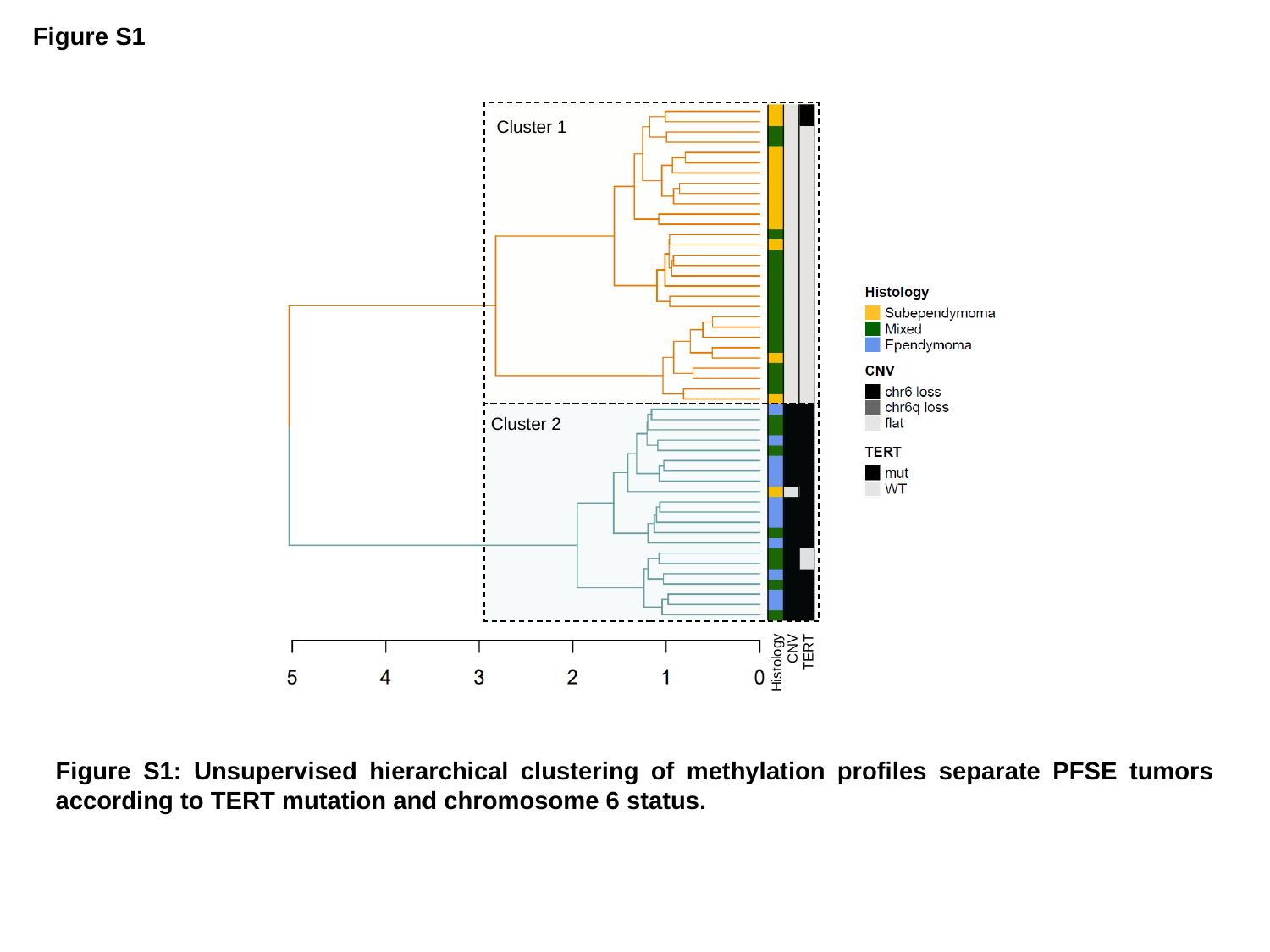

Figure S1
Cluster 1
Cluster 2
Histology
CNV
TERT
Figure S1: Unsupervised hierarchical clustering of methylation profiles separate PFSE tumors according to TERT mutation and chromosome 6 status.

## Slide 2
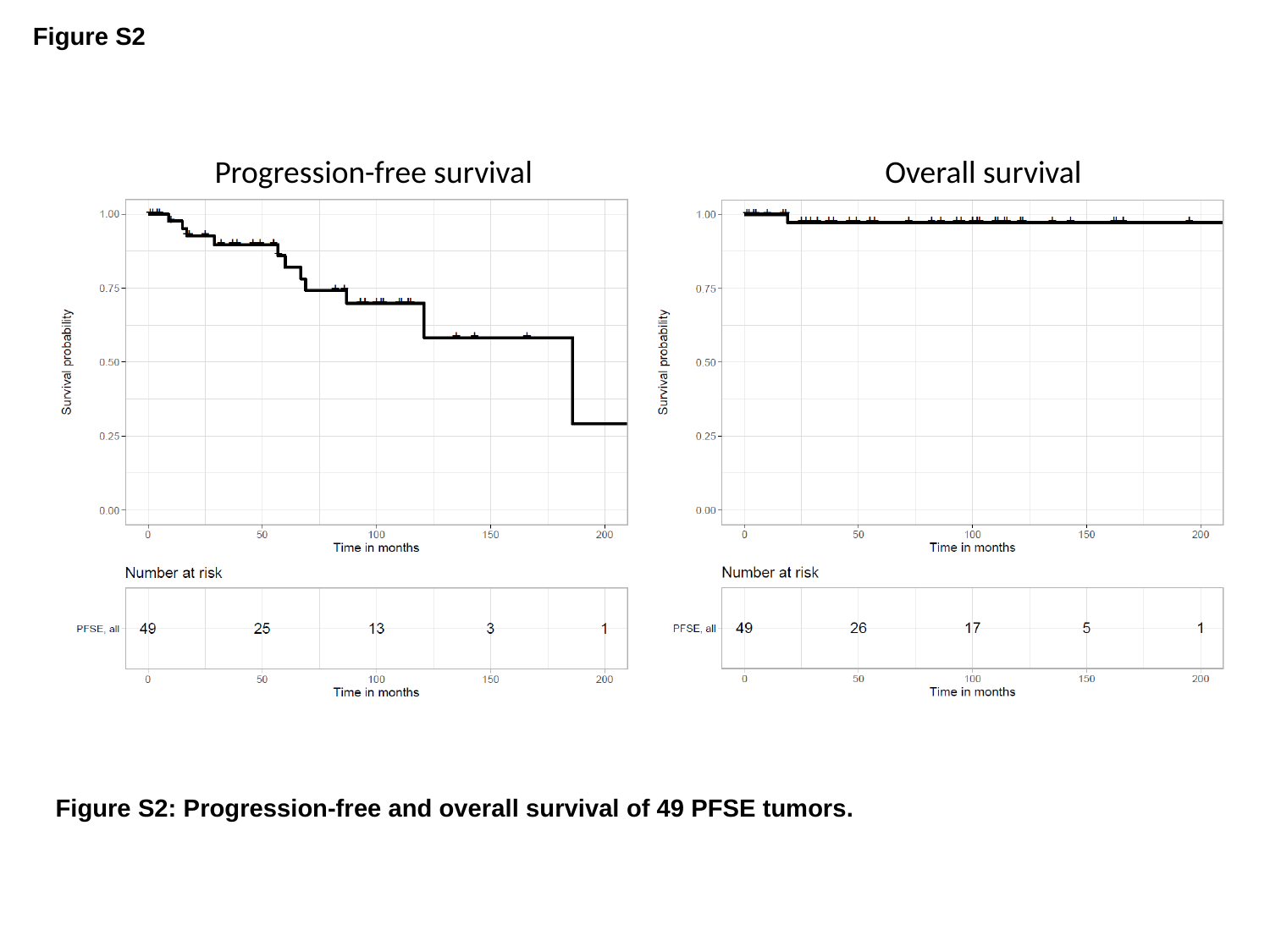

Figure S2
Progression-free survival
Overall survival
Figure S2: Progression-free and overall survival of 49 PFSE tumors.

## Slide 3
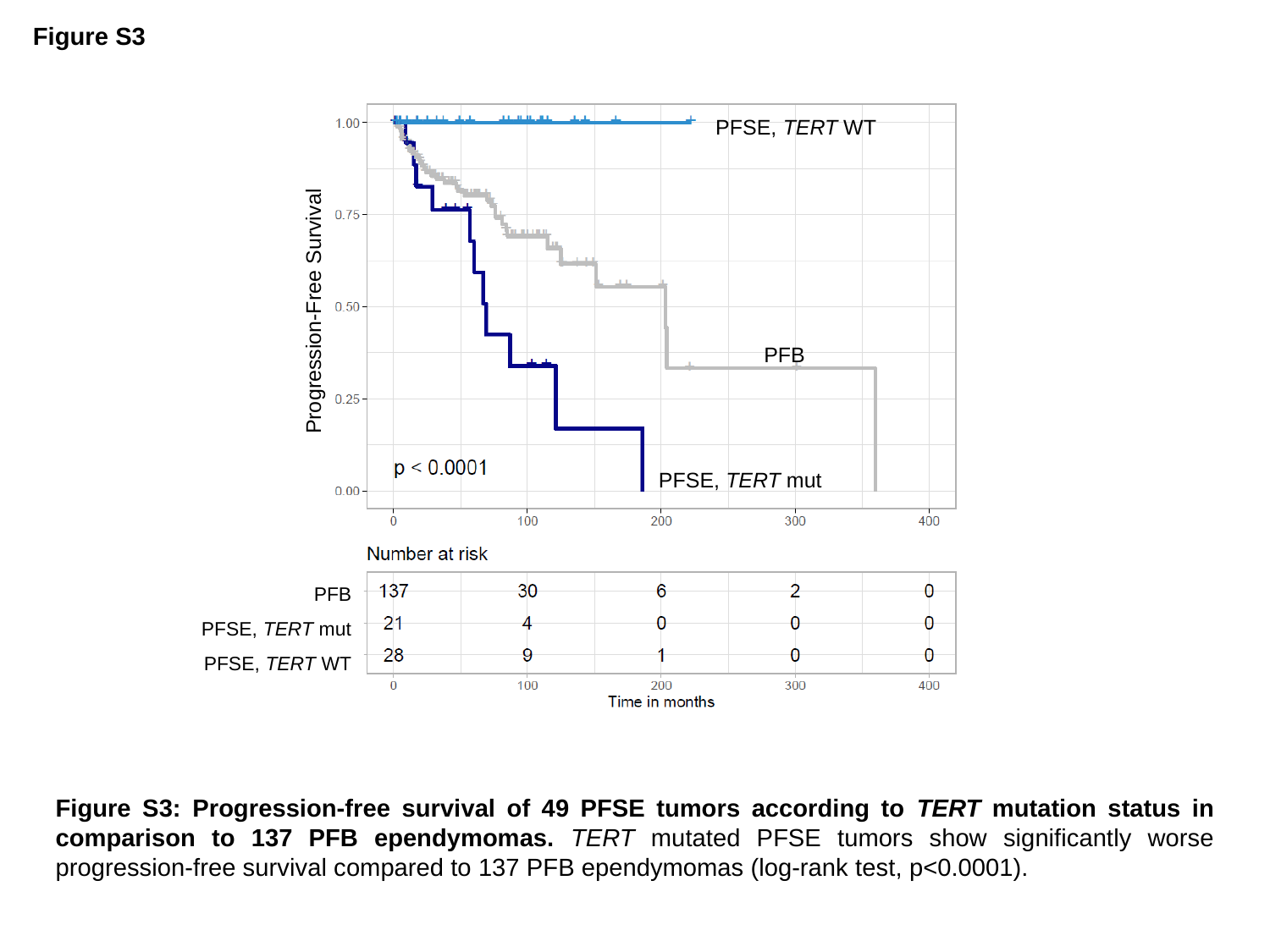

Figure S3
PFSE, TERT WT
Progression-Free Survival
PFB
PFSE, TERT mut
PFB
PFSE, TERT mut
PFSE, TERT WT
Figure S3: Progression-free survival of 49 PFSE tumors according to TERT mutation status in comparison to 137 PFB ependymomas. TERT mutated PFSE tumors show significantly worse progression-free survival compared to 137 PFB ependymomas (log-rank test, p<0.0001).
